# Improving Naloxone Co-prescribing Through Clinical Decision Support

**DOI:** 10.7759/cureus.63919

**Published:** 2024-07-05

**Authors:** Elizabeth Cramer, Ethan Kuperman, Nathan Meyer, James Blum

**Affiliations:** 1 Family Medicine, University of Iowa Hospitals and Clinics, Iowa City, USA; 2 Health Care Information Systems, University of Iowa Hospitals and Clinics, Iowa City, USA; 3 Internal Medicine, University of Iowa Hospitals and Clinics, Iowa City, USA; 4 Anesthesia, University of Iowa Hospitals and Clinics, Iowa City, USA

**Keywords:** applications of health informatics, informatics, opiod overdose, overdose prevention, overdose, opiods, naloxone, prescription opioid overdose deaths, clinical decision tool, clinical decision support system

## Abstract

Background: Despite national guidelines recommending naloxone co-prescription with high-risk medications, rates remain low nationally. This was reflected at our institution with remarkably low naloxone prescribing rates. We sought to determine if a clinical decision support (CDS) tool could increase rates of naloxone co-prescribing with high-risk prescriptions.

Methods: An alert in the electronic health record was triggered upon signing an order for a high-risk opioid medication without a naloxone co-prescription. We examined all opioid prescriptions written by family and general internal medicine practitioners at the University of Iowa Hospitals and Clinics in outpatient encounters between November 30, 2020, and February 28, 2022. Once triggered by a high-risk prescription, the CDS tool had the option to choose an order set with an automatically selected co-prescription for naloxone along with patient instructions automatically added to the patient's after-visit summary (AVS). We examined the monthly percentage of patients receiving Schedule II opioid prescriptions ≥90 morphine milliequivalents (MME)/day who received concurrent naloxone prescriptions in the 12 months before the CDS went live and the three months following go-live.

Results: Concurrent naloxone prescriptions increased from 1.1% in the 12 months prior to implementation in November 2021 to 9.4% (p<0.001) during the post-intervention period across eight family medicine and internal medicine clinics.

Discussion: This single-center quality improvement project with retrospective analysis demonstrates the potential efficacy of a single CDS tool in increasing the rate of naloxone prescription. The impact of such prescribing on overall mortality requires further research.

Conclusions: The CDS tool was easy to implement and improved rates of appropriate naloxone co-prescribing.

## Introduction

In 2021, 107,000 Americans died of an overdose, with nearly 75% attributed to opioids [1]. Naloxone, an opioid antagonist, plays an essential role in evidence-based strategies for decreasing opioid-related fatalities [2,3]. Community-based naloxone distribution programs have been well-examined and show decreased mortality associated with opioid overdose [4]. Other efforts described in the literature have aimed to improve prescribing from the ED, both for individuals at-risk for overdose and for those following an overdose [5,6]. For outpatient prescribing, the Centers for Disease Control and Prevention (CDC) recommends providers follow the CDC Guideline for Prescribing Opioids for Chronic Pain to appropriately prescribe opioids and to consider offering naloxone co-prescription with high-risk medications [7]. Improving naloxone prescribing in the outpatient space is particularly attractive as all efforts will be aimed at the primary prevention of opioid overdose. Previous reports showed that patients who received a naloxone prescription from their primary care provider had 47% less opioid-related emergency room visits six months after naloxone prescription and 63% fewer visits 12 months after, compared to those who did not [8]. Despite this, naloxone prescribing has remained low nationally, co-prescribed with less than 2% of high-dose opioid prescriptions, indicating an opportunity to improve care and save lives [8-10].

One strategy to improve naloxone co-prescribing is through clinical decision support (CDS). CDS utilizes the electronic health record (EHR) to provide clinicians, staff, or patients with knowledge and person-specific information to enhance health and healthcare [11]. The most common format is an automated alert to providers at the time of a desired action. A previous CDS system based in an emergency room setting increased naloxone prescribing from 1.5% of extremely high-risk individuals to 28.7% [6].

Although our institutional co-prescribing was near the national rate, this remained unacceptably low given the opioid epidemic. We aim to improve our institution’s co-prescription rate by 100% through CDS to identify the high-risk population, provide an easy way to prescribe naloxone, and provide patient education.

## Materials and methods

Study setting

We performed this intervention at a university health system utilizing the Epic EHR platform (Verona, WI: Epic Systems Corp.). An interruptive alert known as the Best Practice Advisories (BPA) was the CDS tool selected. BPAs present a notice to the provider and require a response to continue using the system. Our BPA was implemented for physicians and advanced practice providers (APPs) serving in outpatient clinics in the departments of general internal medicine and family medicine. This included eight different clinical spaces spanning central and southeast Iowa. All faculty, resident, and physician extender clinics were included.

Intervention

Starting

In November 2021, the BPA triggered upon signing an order for an opioid medication, benzodiazepine, or muscle relaxant if all six criteria were met (Table 1). A morphine milliequivalents (MME) threshold ≥90 mg/day was chosen for our pilot implementation rather than the CDC-recommended MME of 50 mg/day to minimize alert fatigue and focus on patients felt to be at highest risk for overdose, mimicking other standardized protocols [12].

**Table 1:** Patient inclusion criteria. BPA: Best Practice Advisories; PMH: past medical history; MME: morphine milliequivalents

Required criteria for BPA	
1	Patient without naloxone prescription
2	Appointment status = arrived (so alert will not fire before patient is present)
3	Patient should not have hospice, palliative, or end-of-life condition detected in problem list
4	Patient should not have hospice admission detected in the past year
5	Patient should not have palliative specialty appointment detected in the past year
6	On signing an opioid order, patient has one or more of the following: existing benzodiazepine prescription detected, existing non-benzodiazepine muscle relaxant detected (e.g. baclofen), “opioid use disorder” active on the problem list or in PMH, or “opioid overdose” active on the problem list or in PMH, opioid prescription MME/day threshold ≥90
	OR
	On signing benzodiazepine or muscle relaxant order: patient has opioid prescription signed in the past three months

**Figure 1:**
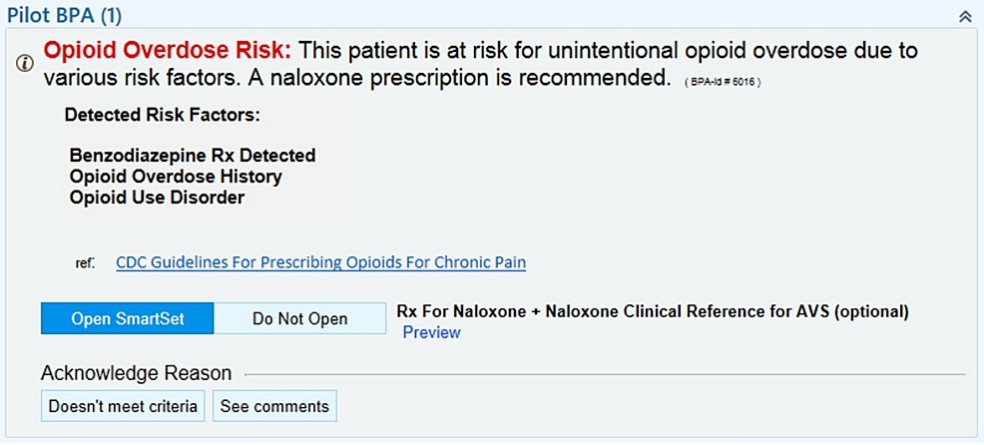
Pilot naloxone BPA pop-up. Epic (Verona, WI: Epic Systems Corp.) AVS: after-visit summary; BPA: Best Practice Advisories

**Figure 2:**
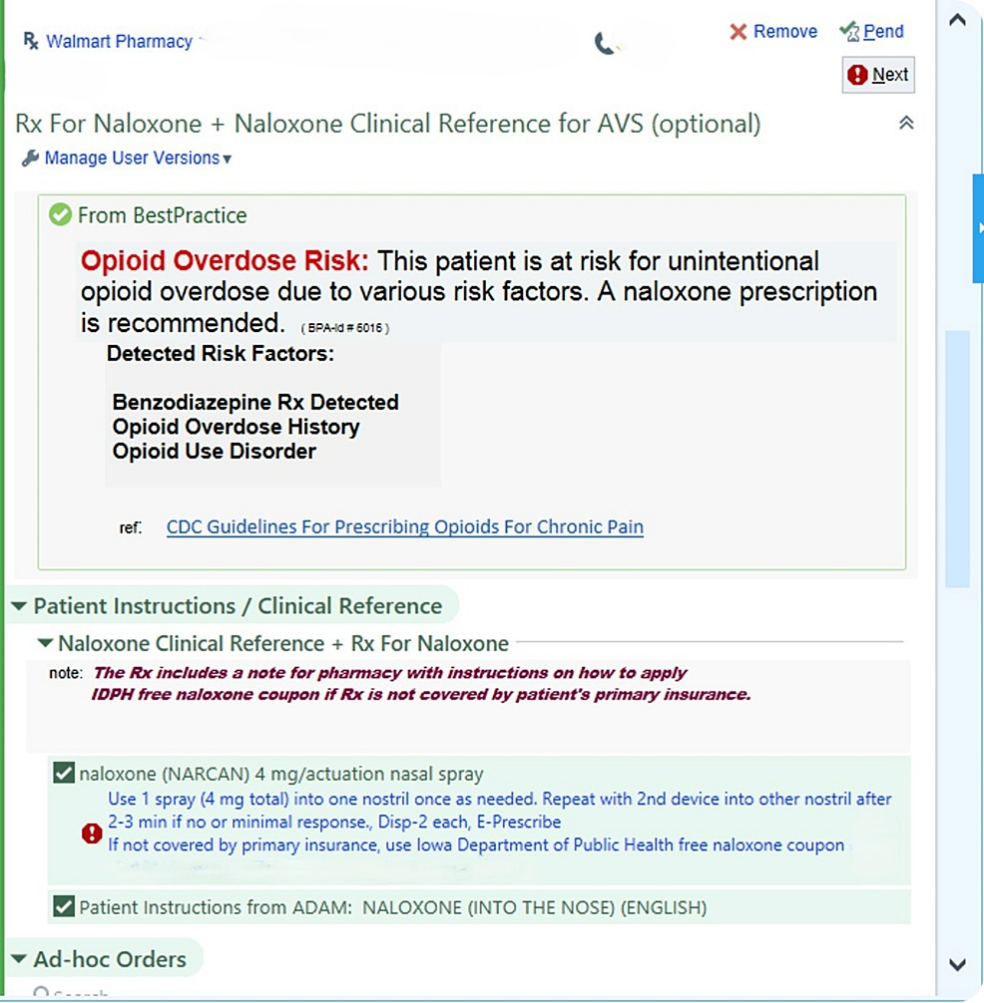
BPA naloxone prescribing options. BPA: Best Practice Advisories; CDC: Centers for Disease Control and Prevention; AVS: after-visit summary

Once presented with the BPA (Figure 1), the provider had the option to choose an order set with an automatically selected co-prescription for naloxone along with patient instructions that would automatically be added to the patient's after-visit summary (AVS) for that clinic encounter (Figure 2). Pre-formatted instructions for the pharmacy were included on the naloxone prescription to ensure that if the patient's insurance did not cover the prescription, patients would get the naloxone at no cost through a state-funded program. Alternatively, the provider could choose an acknowledgment reason for not writing a co-prescription: “Doesn't meet criteria” or “See comments” with a required comment. Both resulted in a 36-hour lockout of the BPA to reduce alert fatigue.

Endpoints

Our primary endpoint was the monthly percentage of patients receiving Schedule II opioid prescriptions ≥90 MME/day who received concurrent naloxone prescriptions. Patients receiving combination agonist/antagonist therapy (e.g., buprenorphine/naloxone), Schedule III or IV opioids (e.g., low-dose codeine), or poor systemic absorption (e.g., loperamide) were excluded from the analysis. Historical prescriptions from external providers reported by patients were excluded from this analysis. We compared those patients who received naloxone vs. those who did not across basic demographic variables, including gender, race, and age. We also compared BPA response between provider specialty (general internal medicine vs. family medicine) and provider background (trainee, APP, and staff physician). Alert performance was characterized by burden (number of alerts), efficiency (naloxone prescriptions per alert), and alert effectiveness (naloxone prescriptions per unique patient). Monthly prescriptions for opioids ≥90 MME/day were tracked as we hypothesized that a reduction may be a secondary benefit of the BPA guidance through alert avoidance. Data were abstracted through the institutional electronic medical record.

Statistical methods

High-dose opioid prescriptions and concurrent naloxone prescribing were analyzed across a 12-month baseline period and a three-month post-intervention period using a p-chart. Categorical variables were compared by chi-square testing. Continuous parametric variables (e.g., age) were analyzed by t-test. Due to high skew, MME/day was compared using Mann-Whitney U test. Analysis was performed in Excel (Redmond, WA: Microsoft Corp.) using the QI Macros Plugin (Denver, CO: KnowWare International Inc.).

## Results

**Figure 3:**
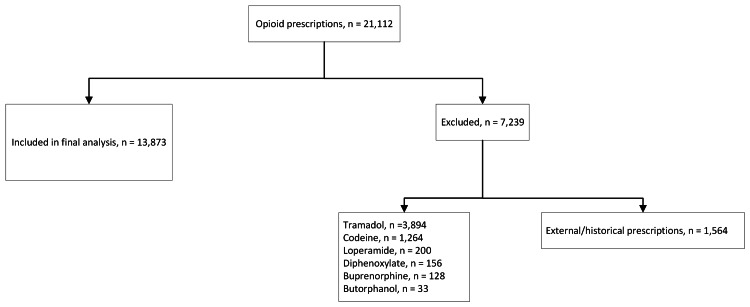
Opioid prescriptions among family medicine and general internal medicine clinics are included for analysis.

Between November 30, 2020, and February 28, 2022, there were 21,112 opioid prescriptions within our family medicine and general internal medicine primary care clinics. Of these, 13,873 met the inclusion criteria (Figure 3). The age, gender, and race of patients receiving opioid prescriptions were similar before and after the intervention (Table 2). However, there were small decreases in the proportion of prescriptions ≥90 MME/day, those from general internal medicine clinics, and those signed by a staff physician.

**Table 2:** Patient demographics of all opioid prescriptions meeting inclusion criteria, before and after BPA implementation. *P-values were calculated using two-sided t-test. **P-values were calculated using chi-square test. ***P-values were calculated using Mann-Whitney U test. MME: morphine milliequivalents; BPA: Best Practice Advisories

Variables	Before (n=11,197)	After (n=2,676)	p-Value
Mean age, years (SD)	57.2 (13.8)	57.0 (14.3)	0.455*
Gender, N female (%)	6,571 (58.7)	1,586 (59.3)	0.583**
Race, N (%)			
White	9,519 (85.0)	2,286 (85.4)	0.700**
Black	900 (8.0)	202 (7.5)	
Other	778 (6.9)	188 (7.0)	
Median MME/day (IQR)	30 (20-60)	30 (20-60)	0.889***
≥90 MME/day, n (%)	956 (8.5)	192 (7.2)	0.021**
Missing data, n (%)	1,750 (15.6)	447 (16.7)	0.171**
Specialty service, n (%)			
Family medicine	6,204 (55.4)	1,551 (58.0)	0.017**
General internal medicine	4,993 (44.6)	1,125 (42.0)	
Provider background, n (%)			
Resident	514 (4.6)	156 (5.8)	0.007**
Advanced practice provider	1,358 (12.1)	344 (12.9)	
Staff physician	9,198 (82.1)	2,157 (80.6)	
Other	127 (1.1)	19 (0.7)	

**Figure 4:**
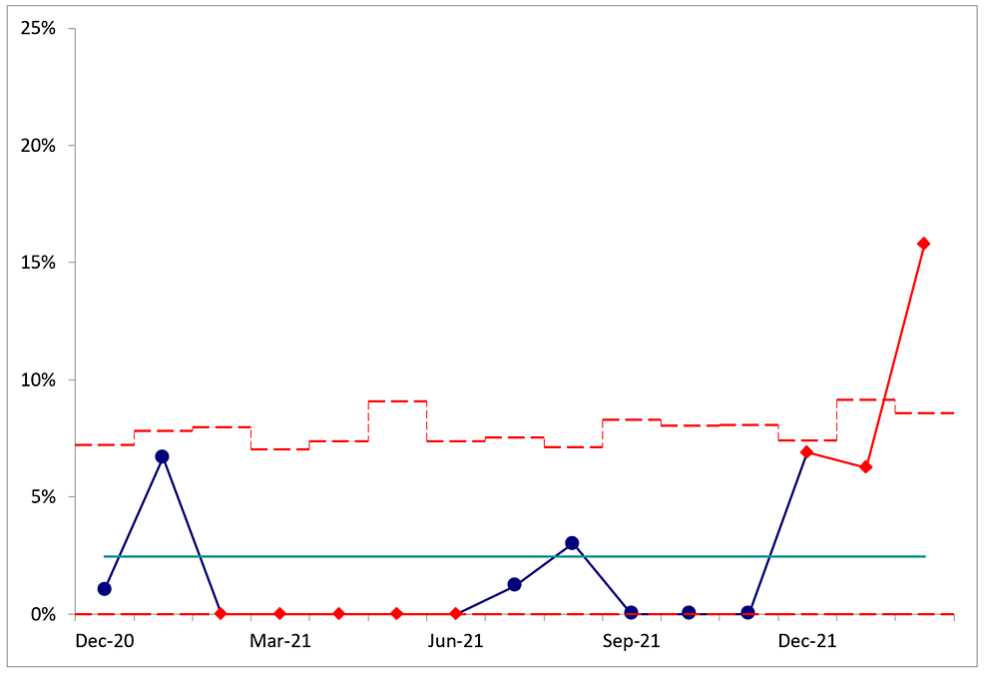
P-chart of naloxone co-prescribing with high-dose (≥90 MME/day) opioid prescriptions. Immediately after intervention in December 2021, special cause variation is observed. Centerline represents the mean performance throughout the study period. Dashed line represents the three-sigma upper confidence limit. MME: morphine milliequivalents

Opioid prescriptions ≥90 MME/day with concurrent naloxone prescriptions increased from 1.1% in the 12 months prior to implementation in November 2021 to 9.4% (p<0.001) during the post-intervention period, an increase of 854% (Figure 4). There were similar increases in naloxone for opioid prescriptions <90 MME/day (1.6-10.2%, p<0.001) and for opioid prescriptions with missing information (0.6-2.7%, p<0.001) (Table 3). There were significant increases in naloxone co-prescribing across all pre-planned subgroups of age, gender, race, specialty service, and provider background.

**Table 3:** Number (percentage of opioid prescriptions in that population) of naloxone co-prescriptions based on patient, provider, and prescription characteristics. P-values represent chi-square comparison before and after the intervention. MME: morphine milliequivalents

Variables	Before	After	p-Value
Age, n (%)			
≤65 years	111 (1.3)	171 (8.5)	<0.001
>65 years	49 (1.8)	67 (10.1)	<0.001
Gender, n (%)			
Female	88 (1.3)	144 (9.1)	<0.001
Male	72 (1.6)	94 (8.6)	<0.001
Race, n (%)			
White	144 (1.5)	202 (8.8)	<0.001
Black	9 (1.0)	23 (11.4)	<0.001
Other	7 (0.9)	13 (6.9)	<0.001
Opioid prescription, n (%)			
<90 MME	140 (1.6)	208 (10.2)	<0.001
≥90 MME	10 (1.0)	18 (9.4)	<0.001
Information missing	10 (0.6)	12 (2.7)	<0.001
Specialty service, n (%)			
Family medicine	59 (1.0)	120 (7.7)	<0.001
General internal medicine	101 (2.0)	118 (10.5)	<0.001
Provider background, n (%)			
Resident	19 (3.7)	14 (9.0)	0.008
Advanced practice provider	4 (0.3)	27 (7.8)	<0.001
Staff physician	136 (1.5)	194 (9.0)	<0.001
Other	1 (0.8)	3 (16)	<0.001

The BPA was triggered 486 times across 383 patients during the post-intervention period. The most common provider responses to the alert were “Do not open” (N=247, 50.8%) and “Open SmartSet” (N=179, 36.8%). Of the latter, 177 resulted in an order being selected for naloxone which represented a significant fraction of the total of 238 naloxone prescriptions across the post-intervention period. Alert efficiency was 36.4% and effectiveness was 46.2%. Provider responses to the BPA varied across levels of training and specialty, with family medicine providers and staff physicians being more likely to order naloxone (Table 4). In contrast, the rate of naloxone orders selected from the BPA was similar across triggering medications, including Schedule III and IV opioids and non-benzodiazepine muscle relaxants.

**Table 4:** Alert responses based on provider characteristics and triggering medication. P-values represent chi-square comparisons for differences in rate of order selection across groups.

Variables	Total alerts (n=486)	Orders selected (n=177)	p-Value
Specialty service, n (%)			
Family medicine	322 (66.3)	128 (72.3)	0.032
General internal medicine	164 (33.7)	49 (27.7)	
Provider background, n (%)			
Resident	51 (10.5)	10 (5.6)	0.025
Advanced practice provider	101 (20.8)	36 (20.3)	
Staff physician	334 (68.7)	131 (74.0)	
Medication triggering alert, n (%)			
Schedule II opioid	183 (37.7)	75 (42.4)	0.328
Other opioid	69 (14.2)	26 (14.7)	
Benzodiazepine	187 (38.5)	62 (35.0)	
Other muscle relaxant	47 (9.7)	14 (7.9)	

## Discussion

Our CDS tool improved rates of naloxone co-prescribing with high-risk opioid prescriptions by 854%, although the absolute rate of co-prescribing remained below 10%. While the alert had the greatest direct effect within the family medicine clinics, naloxone co-prescribing increased across all sites and provider groups as well as all patient demographics.

One possible explanation for prescribing rates remaining low is that the quantity and length of time the narcotic prescription is active are not currently factored into the MME/day equation in our EHR. This mainly affects prescriptions that are written "prn" or "as needed". For example, if a clinician writes a prescription for 5 mg hydrocodone to be taken as needed every 6 hours, but only prescribes five tablets to be used in 30 days, the MME/day is the same as if they had prescribed a quantity of 120 in 30 days because it calculates the maximum amount that could be taken in a day. Other studies have examined ways to calculate MME/day that do take quantity and time into account as this may more accurately reflect the clinician's intentions and what the patient is taking on a daily basis [13]. In this example, MME/day would be lower than what is calculated in our EHR. Nevertheless, calculating it without quantity and time variables gives a higher MME/day and most conservatively and safely describes the maximum a patient could take in a day according to the prescription. 

Our results were similar to those described at the Vanderbilt University Medical Center, with the house-wide implementation of a BPA to promote naloxone prescribing [14]. Our initial rates of naloxone prescribing (1.1%) were higher than those in the Vanderbilt report (0.28%); however, we only implemented our CDS tool in two outpatient departments whereas the Vanderbilt implementation was across the institution. This intervention may have variable efficacy across surgical or subspecialty clinics. Other studies have also shown that a CDS tool can improve naloxone co-prescribing successfully; however, they were studied in the emergency department setting [6].

Our BPA improved the rate of prescribing naloxone to high-risk patients being prescribed opioids. We observed that our proportion of prescriptions with MME/day ≥90 mg decreased as well from 8.5% to 7.2%. Anecdotally, we received feedback from providers that prescribing naloxone to high-risk patients provided another opportunity to discuss the disadvantages of long-term opioid use and was used as a tool to further motivate patients to decrease or stop chronic opioids.

When we examined the breakdown of actions by the role of the BPA, we found that residents were most likely to choose the options of “Do not open” or “See comments” within the BPA, but physician faculty and APPs were both equally likely to prescribe using the BPA, at approximately 40%. Examination of the comments that were left in the comments also reinforces the need for further education. Examples include "has been on chronic narcotics for years," "rare diazepam use," "I have been prescribing same dose of Klonopin for years," "for broken toes," and "I am not prescribing opioids" highlight the need for additional education for naloxone prescribing. A review article showed that targeted provider education described as “academic detailing” can improve naloxone prescribing habits, especially when the education is provided one-on-one, typically between providers and clinical pharmacists [15]. This shows an opportunity for targeted education at our institution underscoring a potential educational need for residents and medical students.

While CDS tools have shown promise to increase rates of co-prescribing of naloxone, other strategies have also shown to be effective at increasing naloxone availability in communities. Efforts to increase naloxone distribution through Opioid Education and Naloxone Distribution (OEND) Programs and through direct dispensation from pharmacies have been found to decrease mortality from opioid overdoses. Iowa has had a state-wide standing order for pharmacists to dispense naloxone since 2016. A statewide study in Massachusetts reported that communities with an OEND had lower mortality rates for opioid overdoses than those without [3]. Abouk et al. found that states allowing pharmacists to independently dispense naloxone to high-risk patients had a 34% decrease in opioid-related mortality [16]. Within states that allowed pharmacist-initiated dispensing, clinical decision support (CDS) systems increased dispensing to high-risk patients, but overall rates remained low [17,18]. For example, one program increased naloxone dispensing by 163% by implementing a three-question patient survey; however, even with this increase, only 35% of eligible patients received prescriptions for naloxone [19]. Another way to increase naloxone availability is through direct community outreach programs where naloxone kits are distributed to at-risk populations. There is evidence that these community programs can be effective in decreasing opioid overdose rates in their communities, but the programs themselves can be costly to implement and maintain [20]. However, according to the CDC, non-prescription opioids were responsible for 79% of opioid deaths in 2021, so it’s possible that naloxone co-prescribing, direct pharmacy dispensation, and community outreach are all needed in order to prevent opioid overdoses related to both prescription and non-prescription opioid overdoses [21].

The limitations of our study include that while we infer that the increase in naloxone prescriptions can be attributed to BPA, we are unable to demonstrate a direct causal relationship between BPA and provider decision-making. It’s possible that a general push from multiple sources may have led to an increase in prescribing. Another limitation was that we were unable to incorporate pharmacy data into our results including the number of naloxone products that were dispensed because of the BPA, and additionally, if any naloxone was dispensed as a part of our state’s Naloxone Dispensing Program without a prescription. Further study on this subject should include ways to estimate lives saved or overdoses prevented because of increases in naloxone prescribing. Finally, we plan to further examine the effect of this BPA over time to ensure that the three-month follow-up was sufficient to determine a change in prescribing behavior.

## Conclusions

Our electronic implementation of a CDS alert improved naloxone prescribing rates. It is a simple intervention that improves patient safety and potentially patient morbidity and mortality. Although the absolute rate of co-prescribing remained below 10%, this BPA helped us identify a group of providers we can target for one-on-one education to improve naloxone prescribing and/or use of the BPA. An additional benefit was a decrease in the proportion of opioids prescribed with MME/day ≥90 mg. Given the success of this BPA in outpatient primary care departments, we will expand this BPA to all outpatient departments and hospital discharges.

## References

[1] (2023). Drug overdose deaths. https://www.cdc.gov/drugoverdose/deaths/index.html.

[2] Visconti AJ, Santos GM, Lemos NP, Burke C, Coffin PO (2015). Opioid overdose deaths in the city and county of San Francisco: prevalence, distribution, and disparities. J Urban Health.

[3] Walley AY, Xuan Z, Hackman HH (2013). Opioid overdose rates and implementation of overdose education and nasal naloxone distribution in Massachusetts: interrupted time series analysis. Br Med J.

[4] Naumann RB, Durrance CP, Ranapurwala SI (2019). Impact of a community-based naloxone distribution program on opioid overdose death rates. Drug Alcohol Depend.

[5] Weiner SG, Carroll AD, Brisbon NM (2022). Evaluating disparities in prescribing of naloxone after emergency department treatment of opioid overdose. J Subst Abuse Treat.

[6] Funke M, Kaplan MC, Glover H (2021). Increasing naloxone prescribing in the emergency department through education and electronic medical record work-aids. Jt Comm J Qual Patient Saf.

[7] Dowell D, Ragan KR, Jones CM, Baldwin GT, Chou R (2022). CDC clinical practice guideline for prescribing opioids for pain - United States, 2022. MMWR Recomm Rep.

[8] Coffin PO, Behar E, Rowe C, Santos GM, Coffa D, Bald M, Vittinghoff E (2016). Nonrandomized intervention study of naloxone coprescription for primary care patients receiving long-term opioid therapy for pain. Ann Intern Med.

[9] (2023). Naloxone. Naloxone.

[10] Follman S, Arora VM, Lyttle C, Moore PQ, Pho MT (2019). Naloxone prescriptions among commercially insured individuals at high risk of opioid overdose. JAMA Netw Open.

[11] Osheroff JA, Teich JM, Middleton B, Steen EB, Wright A, Detmer DE (2007). A roadmap for national action on clinical decision support. J Am Med Inform Assoc.

[12] Jones CM, Compton W, Vythilingam M, Giroir B (2019). Naloxone co-prescribing to patients receiving prescription opioids in the Medicare Part D Program, United States, 2016-2017. JAMA.

[13] Tuan WJ, Sehgal N, Zgierska AE (2021). Using electronic health record's data to assess daily dose of opioids prescribed for outpatients with chronic non-cancer pain. Fam Med Community Health.

[14] Nelson SD, McCoy AB, Rector H (2022). Assessment of a naloxone coprescribing alert for patients at risk of opioid overdose: a quality improvement project. Anesth Analg.

[15] Kulbokas V, Hanson KA, Smart MH, Mandava MR, Lee TA, Pickard AS (2021). Academic detailing interventions for opioid-related outcomes: a scoping review. Drugs Context.

[16] Abouk R, Pacula RL, Powell D (2019). Association between state laws facilitating pharmacy distribution of naloxone and risk of fatal overdose. JAMA Intern Med.

[17] Oliva EM, Christopher ML, Wells D (2017). Opioid overdose education and naloxone distribution: development of the Veterans Health Administration's national program. J Am Pharm Assoc (2003).

[18] Rittel AG, Highland KB, Maneval MS (2022). Development, implementation, and evaluation of a clinical decision support tool to improve naloxone coprescription within Military Health System pharmacies. Am J Health Syst Pharm.

[19] Taylor SR, Chaplin M, Hoots K, Roberts C, Smith K (2020). Effectiveness of implementing a naloxone screening tool in a community pharmacy. Addict Disord Their Treat.

[20] (2024). Opioid overdose. https://www.cdc.gov/drugoverdose/deaths/opioid-overdose.html.

[21] (2024). Naloxone dispensing rate maps. https://www.cdc.gov/drugoverdose/rxrate-maps/naloxone.html.

